# A Genetic Polymorphism in RBP4 Is Associated with Coronary Artery Disease

**DOI:** 10.3390/ijms151222309

**Published:** 2014-12-03

**Authors:** Ke Wan, Jianxun Zhao, Ying Deng, Xi Chen, Qing Zhang, Zhi Zeng, Li Zhang, Yucheng Chen

**Affiliations:** 1 Department of Cardiology, West China Hospital, Sichuan University, Chengdu 610041, China; E-Mails: wankeabm@sina.com (K.W.); zhaojianxun100@gmail.com (J.Z.); cxweirdo@gmail.com (X.C.); qzhang2000cn@yahoo.com (Q.Z.); zengzhi@cd120.com (Z.Z.); 2 National Center for Birth Defects Monitoring, West China Second University Hospital, Sichuan University, Chengdu 610041, China; E-Mail: dengying03@126.com

**Keywords:** *RBP4*, polymorphism, SNP, coronary artery disease (CAD)

## Abstract

Insulin resistance and obesity is influenced by the retinol binding protein 4 (*RBP4*) adipokine. This study aims to determine if genetic polymorphisms in *RBP4* are associated with the risk of coronary artery disease (CAD) in Chinese patients. *RBP4* polymorphisms were analyzed by high resolution melting (HRM) analysis in a case-control study of 392 unrelated CAD patients and 368 controls from China. The Gensini score was used to determine the severity of CAD. The genotypic and allelic frequencies of *RBP4* single-nucleotide polymorphisms were evaluated for associations with CAD and severity of disease. The A allele frequency was significantly higher in CAD case groups compared to control groups (16.7% *vs.* 8.8%) at the *RBP4* rs7094671 locus. Compared to the G allele, this allele was associated with a higher risk of CAD (OR = 2.07 (1.50–2.84)). Polymorphisms at rs7094671 were found to associate with CAD using either a dominant or recessive model (OR, 95% CI: 1.97, 1.38–2.81; 3.81, 1.53–9.51, respectively). Adjusting for sex, history of smoking, serum TC, TG, LDL-c, and HDL-c, the risk of CAD for carriers remained significantly higher in both dominant and recessive models (OR, 95% CI: 1.68, 1.12–2.51; 2.74, 1.00–7.52, respectively). However, this SNP was not significantly associated with severity of CAD using angiographic scores in multivariable linear regression models (*p* = 0.373). The *RBP4* rs7094671 SNP is associated with CAD; however, our results do not indicate that this locus is associated with clinical severity of CAD or the extent of coronary lesions.

## 1. Introduction

Coronary artery disease (CAD) is polygenic and the leading cause of morbidity and mortality worldwide [1]. Traditional risk factors for CAD are well understood and include older age, hypertension, atrial fibrillation, smoking, and diabetes mellitus. However, research indicates that this is a complex disease influenced by both environmental and genetic factors [2]. Recently, a large number of genes have been implicated in contributing to the development of CAD by genetic association studies [3].

In 2005, a study on the genetics of glucose transport protein 4 (GLUT4) identified a new type of adipokine, retinol-binding protein 4 (*RBP4*), as a contributor to insulin resistance [4]. In this study, adipocyte-specific deletion of GLUT4 led to increased *RBP4* expression and the development of insulin resistance. Conversely, when GLUT4 was overexpressed in adipocytes, *RBP4* expression decreased and insulin sensitivity improved. The connection between *RBP4* and insulin resistance was more specifically demonstrated with *RBP4* transgenic mice and by administering recombinant protein to wild-type mice [4]. Subsequent human studies revealed elevated *RBP4* levels in the plasma of subjects with insulin resistance and a positive correlation between *RBP4* expression and the magnitude of insulin resistance [5,6,7,8,9]. Furthermore, strong experimental evidence suggests that *RBP4* is causally involved in the origin of cardio-metabolic diseases [10]. Finally, a child intervention study demonstrated that the loss of fat mass resulted in reduced *RBP4* level that was accompanied by decreases in levels of systemic inflammation and insulin resistance [11].

In addition to its effects on insulin resistance, positive associations have also been documented between *RBP4* and established cerebrovascular disease (CVD) risk factors including, metabolic syndrome [7,12,13], overall/central obesity [14], dyslipidemia [15], and inflammatory markers [16]. Moreover, *RBP4* levels have been positively associated with carotid intima-media thickness, metabolic complications, atherosclerosis [17]. CAD is associated with higher epicardial *RBP4* and lower GLUT4 levels in epicardial and subcutaneous adipose tissue [18]. Recently, we found that the expression of full-length *RBP4* is associated with a three-fold increased risk of CAD in women [19]. Therefore, *RBP4* may play a role in the development of CAD. The aim of this study was to determine whether there is an association between CAD and *RBP4* genetic polymorphisms in a Chinese population. To test this hypothesis, we examined the association between SNPs within the *RBP4* locus and angiographically-defined CAD severity. These results may be helpful for predicting the development of CAD and for implementing early-stage intervention strategies.

## 2. Results

The demographic profile of subjects at the time of blood collection was comparable between the CAD and control groups (Table 1). Subjects with CAD and control subjects were comparable with respect to age. Furthermore, no significant differences were detected between groups in terms of the levels of triglycerides (TG), cholesterol (TC); subjects with CAD have higher levels of low-density cholesterol (LDL-c) and lower levels of high-density cholesterol (HDL-c) total than controls. However, subjects with CAD were more likely to smoke cigarettes (48.9% *vs.* 29.6%) and be hypertensive (50.5% *vs.* 32.0%) compared to the control group.

**Table 1:** Subject demographics.

Characteristic	CAD ( *n* = 392)	Control ( *n* = 368)	*p-*Value
Age (years) *	62 (10)	64 (10)	0.263
Male ^#^	297 (75.7%)	217 (58.9%)	<0.001
Smoking ^#^	192 (48.9%)	109 (29.6%)	<0.001
Hypertension ^#^	198 (50.5%)	118 (32.0%)	<0.001
BMI *	24.9 (3.7)	24.6 (3.6)	0.123
TC (mmol/L) *	2.14 (1.32)	2.14 (1.32)	0.082
TG (mmol/L) *	3.62 (1.60)	3.38 (1.54)	0.410
LDL-c (mmol/L) *	1.38 (0.79)	1.94 (0.90)	<0.001
HDL-c (mmol/L) *	2.17 (1.13)	1.60 (0.90)	<0.001

* Data represent the mean value (standard deviation); ^#^ data represent the number (percentage) of participants in each group.

### 2.1. Distribution of Genotype and Allele Frequencies between CAD Patients and Controls

We estimated linkage disequilibrium (LD) among the three variants by using SHESIS software [20] (see Figure S1). The SNP rs12265684, rs7094671, and rs13376835 were in very mild linkage disequilibrium with each other (r2 < 0.18). Using the Hardy-Weinberg equation to check the genetic distribution within the two subject groups, we noted a genetic balance at the rs12265684 locus (case group, *p* = 0.09; control group, *p* = 0.25). This indicated that the case and control groups were representative of the population and had no selection bias. However, the genotype and allele frequencies at the rs13376835 locus did not conform to the Hardy-Weinberg equilibrium. There was no significant difference between the CAD and control groups in the allele or genotype frequencies at the rs12265684 locus (*p* > 0.05) (Table 2). At the rs13376835 locus, however, there was a difference in genotype frequency (*p* < 0.001) but not in allele frequency between the CAD and control groups (*p* = 0.27). Significant differences were also found between groups at the rs7094671 locus for both genotype (*p* = 0.001) and C/A allele (*p* = 0.001) frequencies. For example, the frequency of the A allele was significantly higher in the case group than in the control group (16.7% *vs.* 8.8%). Therefore, we can conclude that the A allele at rs7094671 is associated with a higher risk of CAD compared to the G allele (OR = 2.07 (1.50–2.84) (Table 2).

**Table 2:** Allele and genotype frequencies in CAD and control groups.

SNP	Alleles (1/2)	Group	Genotypes (Count)			*p*	Alleles		OR (95% CI)	*p*
			11	12	22		1	2		
rs12265684	C/G	Case	331 (84.4%)	61 (15.6%)	0 (0.0%)	0.69	723 (92.2%)	61 (7.8%)	1.43 (0.95–2.16)	0.51
		Control	328 (88.9%)	41 (11.1%)	0 (0.0%)		697 (94.4%)	41 (5.5%)		
rs7094671	G/A	Case	283 (72.2%)	87 (22.2%)	22 (5.6%)	0.00	653 (83.2%)	131 (16.7%)	2.07 (1.50–2.84)	<0.001
		Control	308 (83.7%)	54 (14.7%)	6 (1.6%)		671 (91.1%)	65 (8.8%)		
rs13376835	A/G	Case	298 (76.0%)	94 (24.0%)	0 (0.0%)	0.00	690 (88.0%)	94 (11.9%)	1.40 (1.00–1.96)	0.27
		Control	320 (87.0%)	48 (13.0%)	0 (0.0%)		671 (93.4%)	65 (6.5%)		

### 2.2. Association between rs7094671 Genotype and CAD

Compared with the G allele at the rs7094671 locus, a significant increased risk of CAD was associated with the A allele using both dominant and regression models (OR, 95% CI: 1.97, 1.38–2.81; 3.81, 1.53–9.51; respectively). Multivariate logistic regression was used to further analyze the data by adjusting for gender, sex, history of smoking, serum TC, serum TG, LDL-c levels, and HDL-c levels. After making these adjustments, there was still a significant difference between rs7094671 polymorphic genotypes related to CAD. The risk of CAD for A allele carriers remained high in both dominant and recessive models (OR, 95% CI: 1.68, 1.12–2.51; 2.74, 1.00–7.52; respectively) (Table 3).

**Table 3:** Association between *RBP4* polymorphic genotypes and the risk of CAD.

Model	Control	CAD	OR (95% CI)	*p*-Value	Adjusted Odds Ratio (95% CI) *	Adjusted *p*-Value
AA + GA ^1^	308	283				
GG	60	109	1.97 (1.38–2.81)	0.000	1.68 (1.12–2.51)	0.011
GG + GA ^2^	362	370				
AA	6	22	3.81 (1.53–9.51)	0.004	2.74 (1.00–7.52)	0.050

^1^ Dominant model: (AA + GA)/GG; ^2^ Recessive model: AA/(AA + GA); * Adjusted for age, sex, BMI, hypertension, history of smoking, TG, TC, HDLC, LDLC using the logistic regression model.

### 2.3. Correlation between rs7094671 Genotype and Severity of Coronary Atherosclerosis

To determine the correlation between the rs7094671 genotype and severity of coronary atherosclerosis, we used multivariable linear regression models. After adjusting for age, sex, BMI, hypertension, history of smoking, TG, TC, HDL-c, and LDL-c, the association between the rs7094671 genotype and severity of coronary atherosclerosis was found to be not significant (Gensini *p* = 0.373) (Table 4).

**Table 4:** Multivariate predictors of CAD severity.

Characteristic	Log Gensini (CAD Severity)		*p*-Value
	B	(SE)	
Age (years)	−0.002	0.002	0.339
Sex	0.176	0.560	0.003
BMI	−0.002	0.006	0.710
Hypertension	−0.210	0.041	0.611
Smoking	−0.500	00.48	0.305
TG	−0.021	0.017	0.221
TC	0.031	0.019	0.096
HDL-c	−0.002	0.029	0.944
LDL-c	−0.044	0.024	0.064
rs7094671	0.031	0.034	0.373

## 3. Discussion

In this study, three SNPs were examined in 760 Chinese subjects to reveal any associations between the gene encoding *RBP4* and CAD. We found that subjects in the CAD patient group were more likely to be homozygous for allele A at the *RBP4* rs7094671 locus compared to a control group. In addition, the frequency of the A allele is higher in the CAD group than the control group. According to these results, we conclude that the homozygous AA genotype at the *RBP4* rs7094671 locus is associated with an increased risk of CAD in Chinese populations. While there are records of a GG genotype at the rs13376835 locus in other Chinese population studies, we did not identify any in this study. The sample size and the geographical factors of the subjects in the study may explain why this genotype was not found. The subjects in this study were predominantly from southwestern China, whereas other studies have been based on subject groups from different geographical locations.

The *RBP4* gene is located on chromosome 10 (10q23–q24) near a region linked to increased fasting glucose levels in European Caucasians [21] and type 2 diabetes (T2D) in Mexican-Americans [22]. The gene encodes a protein of 201 amino acids with a molecular mass of 21 kDa [23]. In adipose tissue, *RBP4* is expressed in mature adipocytes. *RBP4* is a retinol (vitamin A) transport protein, and recently Yang et al. found that it can also contribute to systemic insulin resistance [4]. Reduced *RBP4* levels in serum and adipose tissue strongly correlate with subclinical inflammation and pro-inflammatory cytokine levels [24]. *RBP4* impairs insulin signaling in adipocytes indirectly by inducing pro-inflammatory cytokine production from macrophages through Toll-like receptor 4 (TLR4) and c-Jun N-terminal protein kinase (JNK) pathways [25]. In addition, the *RBP4*/retinol complex stimulates JAK2/STAT5 signaling and expression of the suppressor of cytokine signaling 3, which is implicated in insulin resistance [26]. Increased *RBP4* levels cause insulin resistance, in part by activating adipose tissue APCs that induce Th1 polarization and inflammation, and RBP4-induced activation of macrophages and antigen presentation to CD4T Cells are partially dependent on JNK signaling, resulting in increased levels of TNF and IFN-γ and further increase inflammation [27]. *RBP4* was also found to induce *in vitro* inflammation in endothelial cells, by stimulating expression of pro-inflammatory molecules, such as vascular cell adhesion molecule 1 (VCAM-1), E-selectin, intercellular adhesion molecule 1 (ICAM-1), monocyte chemoattractant protein 1 (MCP-1), and interleukin-6 (IL-6) [25]. *RBP4* levels independently predicted early endothelial dysfunction, linking adipose tissue inflammation and subclinical atherosclerosis [28]. A recent prospective trial suggested its value in predicting CAD in a large women cohort during a follow-up period of nine to 16 years [19]. Its levels were significantly elevated in patients with established carotid atherosclerosis and were positively associated with atherosclerosis severity [29]. These studies reveal that increased *RBP4* expression can directly contribute to endothelial inflammation and may influence the development or progression of vascular inflammation during cardiovascular disease and diabetes. *RBP4* has been recently identified as an HDL associated protein; it is demonstrated that in patients with acute coronary syndrome [30]. It may also influence lipid metabolism in morbid obesity where it is associated with an increase in triglyceride levels and contributes to the formation of small HDL, independently of an insulin-resistant state [15]. These findings are in accordance with the premise that systemic *RBP4* levels promote the development of atherosclerosis. In clinical studies, *RBP4* serum concentrations have been shown to be associated with increased levels of systolic blood pressure. Increased serum *RBP4* levels positively correlated with body mass index (BMI) in obese non-diabetic and diabetic subjects. Several *RBP4* gene variants have been identified that associate with the accumulation of adipose tissue, insulin resistance, and T2D [10,31]. This study is the first to identify a specific polymorphism in the gene encoding *RBP4* associated with CAD.

## 4. Material and Methods

### 4.1. Study Population

From July 2006 to July 2014, 392 patients (ages 51.9 ± 12.6 years for women and 54.1 ± 9.2 years for men) admitted to West China Hospital were enrolled the study following a coronary angiography. The diagnosis of CAD was established by coronary angiography: >70% luminal stenosis of one major coronary artery or >50% of the left main coronary artery was considered positive for CAD. The control group consisted of 368 individuals who had no significant coronary obstruction by coronary angiography (ages 42.3 ± 13.1 years for men, 49.5 ± 8.5 years for women). Patients were excluded if they had diabetes mellitus (DM), active inflammatory conditions, an autoimmune disease, malignancies, a known hematological disorder, or used immunosuppressive drugs. Medical history, physical examinations, and clinical laboratory tests were assessed prior to enrolment. Hypertension was defined as SBP ≥140 mmHg or DBP ≥90 mmHg, or the patient was on antihypertensive medication. DM was diagnosed according to one of the following criteria: (1) a history of diagnosed DM and treatment with medications, diet, and/or exercise; (2) non-fasting blood glucose of >11.1 mmol/L; and (3) current use of hypoglycemic therapy to control DM. The study was approved by the Ethics Committee of West China Hospital of Sichuan University. All patients signed an informed consent prior to their inclusion in the study.

### 4.2. Coronary Angiography and Image Interpretation

We utilized Gensini’s scoring system [32]: angiographic stenosis in the range of 0%–25% was scored as 1 point, 25%–50% was scored as 2 points, 50%–75% was scored as 4 points, 75%–90% was scored as 8 points, 90%–99% was scored as 16 points, and total occlusion was scored as 32 points. Each segment was weighted from 0.5 to 5 depending on the functional significance of the area supplied by that segment. These scores were multiplied by the coefficient defined for each coronary artery and segment and the results were summed.

### 4.3. Blood Sampling and Genotyping

Patient blood samples (approximately 5 mL) were drawn directly from the arterial sheath before diagnostic angiography. Genomic DNA was extracted from whole-blood specimens using a commercially available DNA extraction kit (Tiangen Biotech, Beijing, China) according to the manufacturer’s instructions. We utilized the Hap Map database [33] to identify candidate tag single nucleotide polymorphisms (SNPs) at the *RBP4* genetic locus. Common variants (minor allele frequency (MAF) ≥ 0.2) with *r*^2^ ≥ 0.8 in the Hap Map CHB reference panel were selected. We selected three SNPs from the intron of *RBP4*: rs12265684, rs7094671, and rs13376835. Genotyping was performed by high-resolution melt (HRM) analysis, and the primer sequences used are described in Table 5. Primers were designed with Primer5 (Invitrogen, Carlsbad, CA, USA) and synthesized by Introvogen (Shanghai, China). PCR amplification and HRM were performed in the same reaction conditions in a 96-well plate on the LightCycler 480 II. The reaction mixtures contained 1.0 μL purified genomic DNA, 1.0 μL of forward primer and reverse primer, 2.0 μL MgCl_2_, 1.2 μL Master Mix ([High Resolution Melting Master, Roche] and 9.6 μL dH_2_O). Thermo cycling for PCR included one denaturation cycle at 95 °C for 10 min followed by 55 cycles consisting of denaturation at 95 °C for 15 s, annealing at 61 °C for 20 s and extension at 72 °C for 30 s. After the amplification phase, PCR products were denatured at 95 °C for 1 min and cooled to 40 °C for 1 min to form double-stranded DNA. Then, the HRM was performed by gradually increasing the temperature from 65 to 95 °C at a rate of 0.01 °C/s. Melting of the PCR products was monitored by plotting the change in fluorescence that occurred when a double-stranded DNA binding dye was released. The collected data were analyzed using the LightCycler 480 II Gene Scanning software v2.0 (Roche, Basel, Switzerland).

**Table 5:** Primer sequences used for the HRM analysis.

SNP	F/R	Sequence (5'-3')	Size
rs12265684	F	TTTCTCCGACATCTGAGCCCAT	153 bp
	R	GCACAGGTGCCATCGAGGTTC	
rs7094671	F	ATCTCCTCAGCCCCACCTCT	107 bp
	R	GTGGAGCCCCTCTCTTCAACT	
rs13376835	F	GTGGAGCCCCTCTCTTCAACT	131 bp
	R	CGTAATGGGGGTGGAGAGAAT	

### 4.4. Statistical Analysis

Continuous variables are expressed as the mean ± standard deviation (SD), and categorical variables are reported as counts and percentages. To test for differences between groups, analysis of variance (ANOVA) and chi-squared tests were used for continuous and categorical variables, respectively. Logistic regression multivariate analyses were performed to evaluate the odds ratio (OR) for CAD after adjusting for other conventional risk factors. Variables were tested for normality with Kolmogorov-Smirnov statistics and (+1 natural log) transformed for purposes of parametric analyses. Reverse log-transformation was applied to obtain clinically interpretable values. Linear regression models were constructed to test the additive effect of the SNP on CAD phenotypes including severity.

## 5. Conclusions

In conclusion, this study demonstrates that A/A genotype at the *RBP4* rs7094671 locus is associated with CAD in the Han Chinese population. However, the rs7094671 SNP at this *RBP4* risk locus is not associated with the severity of CAD in a population undergoing coronary angiography.

## Limitations

Our study was a single-center trial. Additionally, although our power was sufficient, the sample size was still relatively small, which could have introduced a degree of bias.

## Supplementary Materials

Supplementary materials can be found at http://www.mdpi.com/1422-0067/15/12/22309/s1.
